# Evaluation of Probiotic Potential and Functional Properties of *Lactobacillus* Strains Isolated from Dhan, Traditional Algerian Goat Milk Butter

**DOI:** 10.3390/foods13233781

**Published:** 2024-11-25

**Authors:** Mohamed Cherif Bentahar, Djilali Benabdelmoumene, Véronique Robert, Said Dahmouni, Wasim S. M. Qadi, Zineb Bengharbi, Philippe Langella, Bouasria Benbouziane, Ebtesam Al-Olayan, Esraa Adnan Dawoud Dawoud, Ahmed Mediani

**Affiliations:** 1 Laboratory of Applied Animal Physiology, SNV Faculty, University of Mostaganem, Mostaganem 27000, Algeria; cherif.bentahar@univ-mosta.dz (M.C.B.); djilali.benabdelmoumene@univ-mosta.dz (D.B.);; 2 Institut National de la Recherche Agronomique, Micalis Institute, UMR 1319 MICALIS, AgroParisTech, Université Paris-Saclay, 78350 Jouy-en-Josas, France; 3 Department of Food Science, Faculty of Science and Technology, Universiti Kebangsaan Malaysia, Bangi 43650, Malaysia; qadiwasim@gmail.com; 4 Bioeconomy Laboratory, SNV Faculty, University of Mostaganem, Mostaganem 27000, Algeria; 5 Department of Zoology, College of Science, King Saud University, Riyadh 11451, Saudi Arabia; eolayan@ksu.edu.sa; 6 Faculty of Pharmacy, Universiti Kebangsaan Malaysia, Bangi 43650, Malaysia; 7 Institute of Systems Biology (INBIOSIS), Universiti Kebangsaan Malaysia (UKM), Bangi 43600, Malaysia

**Keywords:** probiotic, lactic acid bacteria, 16S rRNA, traditional butter, biofilms cell, surface hydrophobicity, biological activities

## Abstract

Goat milk butter, locally known as “Dhan”, from the Sfisfa region of Algeria, holds significant cultural and economic value. This study investigates the probiotic properties of lactic acid bacteria (LAB) present in Dhan, focusing particularly on *Lactobacillus* strains. Molecular identification using 16S rRNA revealed a dominance of *Levilactobacillus brevis* and *Lactiplantibacillus plantarum*, forming a substantial part of the bacterial profile. Three LAB isolates (DC01-A, DC04, and DC06) were selected from fresh samples, and rigorous analyses were performed to evaluate their probiotic properties. Safety assessments confirmed the absence of gelatinase, DNase, and haemolytic activities in all isolates. The isolates demonstrated high tolerance to bile salts and acidic conditions, along with the ability to survive simulated gastrointestinal digestion. Notably, strain DC06 exhibited exceptional survival at low pH (1.5) and high bile salt concentrations (0.15–0.3%). All isolates showed substantial growth in MRS medium with 2% phenol, although growth was significantly decreased at 5% phenol. Furthermore, our strains exhibited high adhesion rates to various solvents, demonstrating their potential for strong interaction with cell membranes. Specifically, adhesion to chloroform was observed at 98.26% for DC01-A, 99.30% for DC04, and 99.20% for DC06. With xylene, the adhesion rates were 75.94% for DC01-A, 61.13% for DC04, and 76.52% for DC06. The LAB strains demonstrated impressive growth in ethanol concentrations up to 12%, but their tolerance did not exceed this concentration. They also exhibited robust growth across temperatures from 10 °C to 37 °C, with strains DC04 and DC06 able to proliferate at 45 °C, though none survived at 50 °C. Additionally, the isolates showed significant resistance to oxidative stress induced by hydrogen peroxide (H_2_O_2_) and displayed medium to high autolytic activity, with rates of 50.86%, 37.53%, and 33.42% for DC01-A, DC04, and DC06, respectively. The cell-free supernatant derived from strain DC04 exhibited significant antimicrobial activity against the tested pathogens, while strain DC06 demonstrated moderate antioxidant activity with the highest DPPH scavenging rate at 68.56%, compared to the probiotic reference strain LGG at 61.28%. These collective findings not only suggest the probiotic viability of LAB strains found in Dhan but also highlight the importance of traditional food practises in contributing to health and nutrition. Consequently, this study supports the potential of traditional Dhan butter as a functional food and encourages further exploration of its health benefits.

## 1. Introduction

In recent years, extensive scientific investigation has revealed the critical role that diet plays in shaping overall health and disease prevention [1]. Africa, with its rich tapestry of fermented foods derived from a diverse array of raw materials including crops, cereals, oilseeds, roots, and milk, provides a unique perspective through which to examine this relationship [2,3]. In particular, North African countries share a wealth of traditional foods that have been passed down through generations, and fermented dairy products traditionally consumed in Algeria hold special places [4]. Traditionally, these fermented dairy products were primarily produced in rural areas for local consumption through the spontaneous fermentation of raw milk sourced from local farms [5]. Among the most prominent of these products is Dhan, also referred to as Smen, a fermented butter made from raw milk using empirical methods. Dhan is not only an essential component of the Algerian diet but also a gastronomic heritage that features prominently in many traditional regional dishes and deserves preservation and protection [6]. Traditional fermented foods, whether derived from plants or animal sources, play a crucial role in global dietary habits. These foods not only serve as valuable sources of essential nutrients but also hold significant potential for promoting overall well-being and offering protection against various diseases [7]. Rich in probiotics, traditional fermented foods are recognized for their numerous health benefits [8].

Probiotics are a diverse group of microorganisms that exert beneficial effects on host organisms when administered in adequate amounts [9]. Research has demonstrated that probiotics confer a variety of physiological benefits, including the detoxification of carcinogens, reduction in cholesterol levels, immunomodulation, the alleviation of allergies, the enhancement of nutrient bioavailability, and the mitigation of lactose intolerance [10]. These benefits are attributed to probiotics’ ability to modify the intestinal microbial ecosystem, enhance intestinal barrier function, produce antimicrobial compounds, and modulate immune responses [11]. In poultry, probiotics have been shown to improve intestinal structural integrity and physiological function and promote overall health. Additionally, they have been linked to enhanced growth performance, improved egg and meat quality, and increased food safety [10]. Furthermore, in human studies, probiotics have been associated with reductions in low-density lipoprotein (LDL) cholesterol, total cholesterol, and triglyceride levels, while simultaneously increasing high-density lipoprotein (HDL) cholesterol levels [12]. The development of high-quality probiotic formulations is crucial for meeting the market demand and creating effective products [13]. This process involves selecting safe and functional strains and ensuring consistent efficacy throughout manufacturing; the European Food Safety Authority (EFSA) has identified several LAB strains with Qualified Presumption of Safety (QPS) status, indicating their safety for use in food and feed chains [14]. Research has expanded the range of health-promoting bacteria, leading to the selection and enhancement of novel strains with improved probiotic properties [15]. *Lactobacillus*, a widely utilized probiotic, exhibits various bifunctional properties, that further emphasize the potential of these novel strains [16]. In addition, the use of wild artisanal cultures in metabiotic production offers a unique approach for developing functional products with enhanced probiotic benefits [17].

In recent years, various products have emerged that come from churning butter and blending new ingredients, such as probiotics, flavours, and spices, which will positively impact consumer attraction and engagement. Butter has been demonstrated to be an effective matrix for incorporating probiotics and maintaining their viability through ageing due to its fat protection properties [18]. Additionally, butter may contain probiotics that can enhance the beneficial effects of naturally occurring nutrients, including vitamins, phospholipids, and conjugated linoleic acid [8]. These probiotics can also positively influence the sensory attributes of butter by releasing triglyceride-free fatty acids, thereby enhancing the texture and flavour of the final product [19]. Several studies have suggested the presence of probiotic strains in butter. For instance, the potential of probiotic whey-based beverages and the application of *Lactococcus lactis* in fermented dairy products, including butter, have been highlighted [20]. Similarly, probiotic strains such as *Lacticaseibacillus casei* and *Lactiplantibacillus plantarum*, commonly found in dairy products, have been identified and characterized [21]. Collectively, these findings indicate that butter may indeed contain probiotic strains; however, further research is warranted to substantiate this claim. The objective of this study was to evaluate the probiotic potential of a *Lactobacillus* strain isolated from Dhan butter. To achieve this, a comprehensive series of in vitro tests were conducted to assess the strain’s tolerance to conditions typically encountered in the digestive tract, its antimicrobial activity, adherence capabilities, and essential probiotic properties related to safety.

## 2. Materials and Methods

### 2.1. Sample Collection

Samples of traditional fermented goat milk butter (Dhan) were purchased from local households in the Sfisifa region of Naama, located in Western Algeria (Figure S1). To ensure the integrity of the samples, they were collected aseptically in 100 mL sterile bottles, adhering to rigorous hygiene protocols to prevent cross-contamination. Immediately after collection, the samples were transported to the laboratory in a cool box with ice packs and kept at 6 °C. Microbiological analyses were conducted 24 h post collection [22].

### 2.2. Isolation of Lactobacillus Strains from Goat Milk Butter

To isolate LAB, first, 10 g of butter was homogenized in 90 mL of sterile phosphate-buffered saline (PBS) (NaCl 8 g, KCl 0.2 g, Na_2_HPO_4_ 1.44 g, KH_2_PO_4_ 0.24 g) (Sigma-Aldrich, St. Louis, MO, USA). Next, a serial dilution was performed using a NaCl-peptone solution (0.85%, *w*/*v*—0.1% peptone) (Sigma-Aldrich, St. Louis, MO, USA). Then,100 μL of the sample was evenly spread onto the surface of MRS solid medium for each dilution (Conda Lab, Madrid, Spain) and incubated anaerobically for 72 h. Subsequently, white or beige LAB colonies (2–3 mm diameter) were selected from each plate and transferred to fresh MRS plates for further purification [23]. Following that, the streak method on MRS agar with 0.5% calcium carbonate (CaCO_3_) was used to identify acid-producing bacteria, with LAB isolates distinguished by clear zones around colonies, indicating CaCO_3_ hydrolysis. These isolates were streaked individually on MRS agar for further examination. After purification, the isolates were transfer to MRS liquid medium and centrifuged at 4 °C, 10,000× *g* for 10 min, The resulting pellet was resuspended in MRS broth containing 30% glycerol and subsequently stored at −80 °C. Before use, LAB cultures were sub-cultured in MRS broth twice. Finally, the probiotic reference strain *Lacticaseibacillus rhamnosus LGG* was obtained from INRA, UMR 1319—France, and utilized for preliminary comparative probiotic characterization [24].

### 2.3. Biochemical Characterization of Lactobacillus Isolates

The LAB were characterized using a series of biochemical assays to evaluate their metabolic and enzymatic profiles.

#### 2.3.1. Gram Staining and Catalase Production

Gram staining and catalase production tests were performed. All experiments were performed in triplicate using *S. aureus* ATCC 25923 and LGG as catalase-positive and catalase-negative control strains, respectively [25]. Based on these properties, three strains were selected for further characterization.

#### 2.3.2. Citrate Assay, Aromatic Activity, and Motility

In the citrate assay, Simmons citrate medium was inoculated with a bacterial colony and incubated at 37 °C for 24–48 h. The assay was assessed based on bacterial growth and colour changes, where the development of a blue hue indicated a positive citrate utilization result, signifying the ability of the bacteria to use citrate as a carbon source [26]. For assessing aromatic activity, bacterial strains were inoculated into Clark and Lubs medium (Pasteur Institute-Algiers, Algeria) and incubated at 30 °C for 24 h. Following incubation, the Vosges–Proskauer (VP) reaction was performed by adding VPI and VPII reagents. The presence of a pink ring indicated a positive result for acetoin production, with reaction intensity graded as weak (+), moderate (++), or strong (+++), depending on the colour intensity [27]. The motility of the LAB was evaluated using mannitol motility medium, where bacterial colonies were incubated at 37 °C for 48 h. Motility was assessed based on bacterial growth patterns, with non-motile strains showing limited growth around the inoculation site, while motile strains exhibited diffused growth throughout the medium [26].

#### 2.3.3. Glucose Fermentation Test

A glucose fermentation test was performed as outlined by Gupta et al. [28]. Bacterial cultures were grown in MRS broth (Conda Lab, Madrid, Spain) for 18 h and harvested by centrifugation at 6000 rpm for 15 min, and the pellets were washed twice with PBS buffer before resuspension. Then, 500 µL of the bacterial suspension in PBS was added to modified MRS broth containing 1% glucose and 0.5% phenol red, with Durham tubes inserted. The broth was incubated at 37 °C for 24 h. Acid production from Hom fermentation was indicated by a colour change from red to yellow, while hetero-fermentation was detected by gas formation in the Durham tube and a colour change.

### 2.4. Identification of Lactobacillus Isolates

#### 2.4.1. Phenotypic Identification Using API 50 CHL Kit Assay

The phenotypic identification of the LAB isolates was carried out using the API 50 CHL analysis kit (Bio Merieux, S.A., Marcy l’Etoile, France), which evaluates the biochemical properties of the isolates for precise identification [29]. Colour changes were assessed according to the manufacturer’s instructions. The obtained results were then compared with the BacDive database (https://bacdive.dsmz.de/api-test-finder accessed on 10 June 2024) to confirm the identity of the LAB isolates. This method provides a reliable approach to characterize LAB based on their metabolic capabilities and facilitates the identification of specific strains for further investigation.

#### 2.4.2. Molecular Identification

Putative *Lactobacillus* isolates were identified at the species level through 16S ribosomal RNA (rRNA) sequencing. Genomic DNA was extracted following the method previously described by Boucard et al. [30], with some modifications. Briefly, cells from a single fresh colony were lysed using 0.1 mm glass beads (Retsch, Haan, Germany) in a 500 µL suspension of 10^10^ *Lactobacilli* and 0.1 g beads. The lysis was performed using the Precellys Evolution homogeniser (Bertin Instruments, Montigny-le-Bretonneux, France) with two 15 s cycles at 5800× *g*, separated by a 30 s rest. Following cell disruption, the debris were removed by centrifugation at 20,000× *g* for 10 min at 20 °C, and the DNA was subsequently collected from the supernatant.

#### 2.4.3. Amplification of 16S rRNA

The amplification of 16S rRNA was conducted using two approaches, generating 1.5 kb DNA fragments using the primers J4 (5′-ACGGCTACCTTGTTACGACTT-3′) and J7 (5′-AGAGTTTGATCCTGGCTCAG-3′) (Eurofins, Luxembourg), which target nearly the full length of the 16S rRNA gene. PCR amplification was performed using a SimpliAmp Thermal Cycler (Applied Biosystems, Foster City, CA, USA) [31]. The reaction mixture consisted of 0.5 µL of Green Buffer 10X (Jena Bioscience, Jena, Germany), 0.5 µL of dNTPs Mix 10 mM (Sigma-Aldrich, St. Louis, MI, USA), and 0.1 µL of DreamTaq Green PCR Master Mix (Thermo Fisher Scientific, Waltham, MA, USA). Additionally, for 1 µL of DNA template (50 ng) and 1 µL of each primer (10 µM), the final volume was adjusted to 25 µL using 20.9 µL nuclease-free water. The amplification protocol included an initial denaturation step at 95 °C for 15 min, followed by 38 cycles of denaturation at 95 °C for 30 s, annealing at 52 °C for 30 s, and extension at 72 °C for 1 min and 30 s. A final extension step was performed at 72 °C for 10 min to ensure complete DNA synthesis.

#### 2.4.4. Sequencing and Phylogenetic Analysis

All DNA sequences were confirmed through 16S rRNA gene sequencing (Eurofins MWG Operon, Ebersberg, Germany) using universal primers J4 and J7 [32]. The resulting sequences were analyzed using the Unipro UGENE program and then subjected to BLAST analysis via the NCBI database. To determine the similarity of the sequences to registered strains, they were compared with entries in the EzTaxon genome database. The 16S rRNA gene sequences of the identified LAB strains were subsequently submitted to the NCBI GenBank. Sequence alignment was carried out using ClustalW within the MEGA 11 software, and a phylogenetic tree was constructed using the neighbour-joining method with 1000 bootstrap replicates. Isolates were assigned to species based on high sequence similarity and clear phylogenetic clustering [33].

### 2.5. Biosafety Testing of Lactobacillus Strains

#### 2.5.1. Deoxyribonuclease (DNase) Test

DNase activity was conducted by inoculating 1 μL aliquots of overnight cultures onto DNase methyl green agar medium (HiMedia Laboratories, Pvt. Ltd., Mumbai, India). Isolates were incubated at 37 °C for 48 h. Positive DNase activity was indicated by the formation of distinct clear halos around the bacterial colonies, signifying the degradation of DNA [34]. This test is essential for evaluating the safety profile of LAB strains, as the absence of DNase activity is generally considered a favourable trait for probiotics.

#### 2.5.2. Haemolytic Activity Test

Haemolytic activity was evaluated following the method described by Xu et al. [35] with minor modifications. Overnight cultures of *Lactobacilli* strains (log 8 CFU/mL) were spotted (10 μL) onto Columbia Agar plates (BioMérieux^®^, Craponne, France) supplemented with 5% (*v*/*v*) defibrinated sheep blood. The plates were incubated at 37 °C for 48 h, after which haemolytic reactions were evaluated by observing the zones surrounding the bacterial colonies. A clear zone around the colonies indicated complete haemolysis (β-haemolysis), a green zone indicated partial haemolysis (α-haemolysis), and the absence of a zone signified no haemolytic activity (γ-haemolysis). The experiment was performed in triplicate and repeated twice independently to ensure reliability.

#### 2.5.3. Production of Gelatinase

Gelatinase production was evaluated by inoculating the bacterial isolates into nutrient gelatin tubes containing a medium composed of peptone (5 g/L), meat extract (3 g/L), and gelatin (120 g/L) (Himedia, Mumbai, India). The tubes were incubated at 30 °C for 7–14 days. The medium was inspected daily for liquefaction. Sustained liquefaction, even after the tubes were cooled for 1 h at 4 °C, was considered a positive indicator of gelatinase activity and the enzymatic hydrolysis of gelatine [36].

#### 2.5.4. Antibiotic Susceptibility

Antibiotic sensitivity was evaluated using the Kirby–Bauer disc diffusion method on MRS agar [37]. Bacterial cultures were spread on freshly prepared MRS agar plates, and antibiotic discs were evenly distributed across the surface. The plates were then incubated at 37 °C for 24–48 h, and the susceptibilities were assessed based on the zone surrounding the disc. The antibiotics used in this study are detailed in Table 1 (Sialchim—Algeria).

### 2.6. Strain Characteristics Associated with Probiotic Potential

#### 2.6.1. Tolerance to Bile Salt

Bile salt resistance was assessed according to the method described in [38]. A 250 μL aliquot of the bacterial suspension (8 log CFU/mL) was inoculated into 5 mL of sterile MRS broth and incubated at 37 °C for 24 h. After incubation, the bacterial cells were harvested by centrifugation (10,000× *g*, 10 min, 4 °C), washed twice in phosphate-buffered saline (PBS), and resuspended in the same buffer. Subsequently, 100 μL of the bacterial suspension was added to 900 µL of bile salt solutions at concentrations of 0.15, 0.30, and 0.50% (*w*/*v*), as well as control solutions (without bile salts). The mixtures were incubated at 37 °C for 2 h. Following incubation, the total viable count was determined using the pour plate method, and the bile tolerance survivability percentage was determined using the following formula:

(1) $$\%\mathrm{Survivability}=\left[\frac{{bile}_{\left(CFU/mL\right)}}{{control}_{\left(CFU/mL\right)}}\right]\times 100$$

#### 2.6.2. Sodium Chloride (NaCl) Tolerance Test

The NaCl resistance of the bacterial strains was evaluated by inoculating cultures (2%) of each culture into MRS broth (10 mL) with different NaCl concentrations (2, 3, 4, 6.4, and 10%), where growth in standard MRS broths without NaCl supplementation was used as a positive control. The cultures were then incubated at 37 °C for 7 days. Bacterial growth was monitored by visual assessment of turbidity in the medium. Strains exhibiting visible growth were recorded as ‘+’, while those with no observable growth were denoted as ‘−’ [39].

#### 2.6.3. pH Resistance

The ability of the bacterial strains to survive and grow under extreme pH conditions was evaluated by assessing their resistance to acidic environments. A 2% bacterial culture was added to 10 mL of MRS broth, which had been adjusted to pH levels of 1.5, 3, and 5 using 1.0 M HCl. Sterile MRS broths with adjusted pH were used as a negative control, and growth in standard MRS broths was used as a positive control. The viability of cells was determined by assessing bacterial growth after 24 and 48 h of incubation at 37 °C. The resistance of the isolates was determined by observing whether the media exhibited turbidity or remained clear [39].

#### 2.6.4. Phenol Resistance

As a result, phenol resistance is a critical trait for the survival of probiotics in the gastrointestinal tract. To assess phenol resistance, 2% of each bacterial culture was inoculated into 10 mL MRS broth containing 0.2 and 0.5% phenol, alongside a control without phenol. After incubation at 37 °C for 24 and 48 h, bacterial growth was evaluated by plating on MRS agar. The presence or absence of colonies in phenol-supplemented media indicated the level of phenol resistance [40].

#### 2.6.5. Auto-Aggregation

The auto-aggregation ability of the LAB isolates was assessed using the method described by Ramos et al. [41], with slight modifications. Activated bacterial cultures were harvested by centrifugation at 5000× *g* for 15 min and washed twice with PBS. The cell pellets were resuspended in PBS to achieve a viable cell count of 8 log CFU/mL. The suspension was mixed thoroughly using a vortex mixer. A total of 200 μL of the suspension was combined with 1800 μL of PBS in a microtiter plate, and the OD was measured at 600 nm (A0). The same process was repeated after 1, 2, and 24 h (At). The auto-aggregation percentage was determined using the following formula:

(2) $$Auto-\mathrm{aggregation}\%=\left[\frac{A_{0}-A_{t}}{A_{0}}\right]\times 100$$

where A_t_ stands for the absorbance after 1, 2, and 24 h.

#### 2.6.6. Bacterial Adhesion to Hydrocarbons (BATH)

The hydrophobicity of LAB strains were evaluated through adhesion to hydrocarbons, according to the methods described by Martiz et al. [42], with minor modifications. Three different solvents were tested in this study: p-xylene, a nonpolar solvent (Merck, Darmstadt, Germany); ethyl acetate, a monopolar and basic solvent (Merck, Darmstadt, Germany); and chloroform, a monopolar and acidic solvent (Merck, Darmstadt, Germany). During the early logarithmic growth phase, cells from each strain were harvested by centrifugation at 8000 rpm for 10 min, then washed twice with PBS, and resuspended in the same buffer to achieve an absorbance of approximately 1.0 at 600 nm. Then, a volume of 1 mL of hydrocarbon solvent was added to 3 mL of the cell suspension. The mixture was vigorously vortexed for 2 min, and the phases were allowed to separate for 1 h at 37 °C. After phase separation, the aqueous phase was carefully transferred to a cuvette, and its absorbance at 600 nm was measured. Hydrophobicity (H%) was expressed as the percentage decrease in absorbance at 600 nm after mixing according to the following formula:

(3) $$\mathrm{Hydrophobicity}\%=\left[\frac{A_{0}-A_{t}}{A_{0}}\right]\times 100$$

where A_0_ and A_t_ are the absorbance values of the aqueous phase before and after contact with the solvent, respectively.

#### 2.6.7. Biofilm Formation Assay

Biofilm formation was assessed using a modified glass surface method following the protocol of Fatima et al. [43]. Briefly, 500 μL of bacterial suspension with an OD₆₀₀ of 0.1 ± 0.01 was inoculated into 10 mL of tryptone soy broth (TSB) supplemented with 1% (*w*/*v*) sucrose in sterile glass tubes. The tubes were incubated at 37 °C angle for 48 h to facilitate biofilm formation, and the sterile TSB tube was used as control. Following incubation, the TSB medium was carefully discarded, and the tubes were aseptically washed three times with PBS pH 7.4 to remove any non-adherent bacteria. The adherent cells were then fixed by adding 3 mL of 99% methanol, and the tubes were allowed to rest for 15 min. The methanol was then removed, and the tubes were allowed to air dry. To assess biofilm formation, 0.3 mL of 0.1% crystal violet solution was added to each tube and incubated for 5–6 min. Excess dye was removed by rinsing with running tap water, and the tubes were inverted to dry. Biofilm adhesion levels were quantified on a scale from 0 (no adhesion) to 4 (strong adhesion).

### 2.7. Characterization of Lactobacillus Strains

The technological potential of the LAB isolates was assessed in vitro through a series of evaluations.

#### 2.7.1. Alcohol Resistance Assay

To evaluate alcohol resistance, bacterial cultures were prepared with an OD₆₀₀ of 0.5 ± 0.01 and inoculated into MRS broth supplemented with ethanol at final concentrations of 3, 6, 12, and 15% (*v*/*v*). The cultures were incubated at 30 °C for 24–48 h. As a control, bacterial cultures were inoculated into respective media without ethanol. Growth was evaluated through the visual observation of turbidity, with growth levels categorized as follows: maximum growth (+++), high growth (++), weak growth (+), and no growth (−) [44].

#### 2.7.2. Hydrogen Peroxide (H_2_O_2_) Resistance Assay

The tolerance of activated cultures to H_2_O_2_ was assessed using the following procedure. The cultures were diluted to a concentration of 1% in MRS broth containing 0.4, 0.7, and 1.0 mM H_2_O_2_ (30%). The cultures were then incubated at 37 °C for 8 h. Cell growth was quantified spectrophotometrically at 600 nm, and the results were reported as optical density values [45].

#### 2.7.3. Growth Evaluation 

The ability of the LAB to thrive at different temperatures was assessed using a modified procedure described by Badis et al. [46]. Bacterial cultures with an OD₆₀₀ of 0.5 ± 0.01 were inoculated into MRS broth and incubated at various temperatures (6, 10, 25, 37, 45, and 50 °C) for 5 days. Growth was determined by observing turbidity in the medium, indicating the strain’s adaptability to temperature variations.

#### 2.7.4. Autolytic Activity Assay

The autolytic activity of the strains was evaluated according to the method described by Barzegar et al. [47], with minor modifications. Overnight cultures grown in MRS broth were harvested, washed twice in PBS, and resuspended in the same buffer to an OD_600nm_ of 0.6–0.8. The cultures were incubated at 37 °C up to 24 h. The degree of autolysis was calculated using the following equation:

(4) $$\mathrm{Autolysis}\%=\left[\frac{A_{0}-A_{24}}{A_{0}}\right]\times 100$$

where A_0_ is the initial absorbance, and A_24_ is the absorbance measured after 24 h of incubation.

### 2.8. Biological Activities of Lactobacillus Strains

#### 2.8.1. Antibacterial Activity Assay

Antibacterial activity was determined according to the protocol in [48], with minor changes. In total, 100 μL of overnight-cultured LAB with an OD_600_ of 0.8 was incorporated into 20 mL of MRS agar. The inoculated agar was then poured into a Petri dish and incubated for 7 days at 37 °C under anaerobic conditions to promote the production of antimicrobial metabolites. The bacterial strains *Staphylococcus aureus* ATCC25923, *Escherichia coli* ATCC10536, and *Pseudomonas aeruginosa* ATCC27853 (obtained from Pasteur Institute—Algeria) were used for the antibacterial test. These strains were cultured in 5 mL tryptic soy broth (TSB) at 37 °C for 24 h and adjusted to an OD600 of 0.08. Subsequently, 100 μL of the prepared bacterial suspension was spread onto Müller–Hinton agar plates using the spread plate technique. Immediately after spreading the bacterial suspension, two LAB agar discs with a diameter of 5 mm were cut and placed on the pathogen plates. The plates were then incubated at 37 °C for a further 24 h. For the negative control, 5 mm discs of MRS agar without LAB were used. After incubation, the presence of clear inhibition zones around the discs suggested antibacterial activity. The diameters of these zones were measured in mm.

#### 2.8.2. Scavenging of 2,2-Diphenyl-β-Picrylhydrazyl (DPPH) Free Radicals

The DPPH radical scavenging activity of LAB was assessed using a modified method described by Xing et al. [49]. The pH of the overnight culture was adjusted to 5.5 with 1 M NaOH, followed by centrifugation at 8000 rpm for 10 min to separate the cells. The resulting supernatant was collected and passed through a 0.2 µm syringe filter to ensure the complete removal of any residual bacterial cells. A 0.1 mM fresh DPPH solution (Riedel-de Haën, Sigma-Aldrich, Seelze, Germany) was prepared in ethanol, and 1 mL of this solution was combined with 0.5 mL of cell-free supernatant (CFS). The mixture was then vigorously shaken and incubated in the dark for 30 min. The DPPH radical scavenging activity was measured at 517 nm using a spectrophotometer, with LGG CFS used as a positive control. The percentage of DPPH radical scavenging activity was calculated using the following equation:

(5) $$\mathrm{DPPH}\mathrm{scavenging}\%=\left[\left(A_{1}-A_{2}\right)/A_{0}\right]\times 100$$

where A_0_ = absorbance of control, A_1_ = absorbance of CFS, and A_2_ = absorbance without DPPH, and the broth was taken as the reference.

### 2.9. Statistical Analysis

Data were statistically analyzed using SAS software for Windows (version 8.0; SAS Institute, Cary, NC, USA). A one-way analysis of variance (ANOVA) was conducted, and the Student–Newman–Keuls test was applied to assess mean differences when significant at α = 0.05. Experimental data are presented as the mean ± standard deviation (SD), with each trial conducted in triplicate.

## 3. Results and Discussion

### 3.1. Preliminary Identification of Lactobacillus Isolates

Twelve isolates were identified as Gram-positive, catalase-negative, and non-motile, classifying them as a presumptive LAB. Among these, three isolates were selected for further investigation (Table 2). These isolates exhibited robust growth in MRS broth under both aerobic and anaerobic conditions, with no evidence of aromatic activity or citrate utilization. However, it is important to note that not all LAB isolates possess these characteristics, as previously reported for LAB isolated from camel and cow milk, which also showed a lack of citrate utilization and diacetyl production [29]. These findings are consistent with those reported by Hadef et al. [8], who reported a predominance of LAB in raw goat milk.

Gas production was assessed as a functional parameter to classify the isolates as either homo- or heterofermentative, which is crucial for selecting suitable food matrices for potential probiotic strains. All strains studied demonstrated gas production from glucose (Table 2), indicating a heterofermentative metabolism. These results align with previous research on LAB characteristics [25]. While many *Lactobacillus* species are homofermentative, producing no gas from glucose, heterofermentative strains are also of significant interest. For instance, homofermentative species like *Lactobacillus gasseri*, which colonizes human mucosal surfaces and confers health benefits, are regarded as promising probiotics [50]. Other species, including *L. acidophilus*, *L. crispatus*, *L. amylovorus*, *L. gallinarum*, and *L. johnsonii*, have also been identified as potential probiotic strains [47]. In contrast, de Almeida Júnior et al. [51] reported that 12% of potential LAB isolates from goat milk exhibited gas production. The use of heterofermentative strains in food production is complex, offering both technological benefits and potential drawbacks. While gas production by *Lactobacillus* is beneficial in the production of certain dairy products, such as kefir and some cheeses, it may be undesirable in others. Further investigation into the metabolic pathways and fermentation abilities of these LAB isolates could provide valuable insights into their potential applications.

### 3.2. Phenotypic Identification

All selected LAB isolates in this study were identified as belonging to the genus *Lactobacillus* (Table 3), further classified into the genera *Levilactobacillus* and *Lactiplantibacillus* following the taxonomy proposed by Zheng et al. [52]. According to Galushko and Kuever [25], these groups are part of LAB. The dominance of these genera is associated with their key roles in fermentation, particularly their ability to acidify the environment and produce antimicrobial metabolites that contribute to food preservation and safety [53]. The prevalence of *Lactobacilli* in fermented product can be attributed to the specific processing method, which involves the acidification of milk for 24 h before starting fat separation. Acidified milk creates a selective environment that favours the growth of acid-tolerant microbial flora. Additionally, the fat separation process occurs in a cooking chamber with anaerobic conditions, which significantly limits oxygen exposure, thereby enhancing the growth of LAB [6].

### 3.3. Identification of Lactobacillus Strains by 16S rRNA Sequencing

Molecular techniques are essential for bacterial identification, offering greater accuracy for identifying LAB compared to traditional phenotypic approaches [54]. Recent taxonomic revisions within the genus *Lactobacillus* have led to the establishment of 23 new genera, including *Lacticaseibacillus*, *Lentilactobacillus*, and *Lactiplantibacillus*, as outlined by Zheng et al. [52] through a polyphasic approach that combines both phenotypic and genotypic methods. In this study, we amplified and sequenced the 16S rRNA gene from the total genomic DNA of selected LAB isolates to determine their taxonomic identities. The obtained sequences demonstrated high similarity, exceeding 99 to 99.93% when compared to sequences available in GenBank (Table 4). The 16S rRNA gene sequencing results confirmed that two of the identified bacterial strains belonged to the genus *Levilactobacillus*, while one strain was classified as *Lactiplantibacillus*. This taxonomic assignment was further verified through the EzBiocloud (Table 5). Additionally, the 16S rDNA sequences obtained from this study have been submitted to GenBank with the following accession numbers: DC01-A (PP709510), DC04 (PQ205308), and DC06 (PP709364) (Figure S2). To further validate the taxonomic placement, a phylogenetic tree was constructed using the neighbour-joining method based on evolutionary distances [55]. The analysis incorporated reference strains from diverse sources, confirming that all isolates were clustered within the *Lactobacillaceae* family.

### 3.4. Biosafety Evaluation of Lactobacillus Strains

#### 3.4.1. Determination of Gelatinase, DNase, and Haemolytic Activities

**Figure 1:**
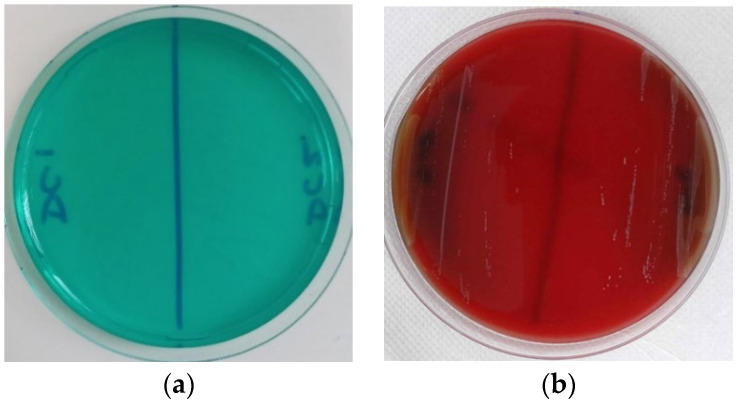
Biosafety evaluation of *Lactobacillus* strains. (**a**) DNase activity and (**b**) haemolytic activity.

Assessing the biosafety of probiotic strains is critical, particularly in terms of potential virulence factors such as DNase production. DNase evades the host’s innate immune response by degrading extracellular neutrophil traps and hydrolysing human DNA, thereby disrupting protein synthesis [56]. This enzyme promotes the growth of pathogens by increasing the availability of nucleotides through DNA hydrolysis and facilitates the spread of pathogens. Furthermore, DNase suppresses both the bactericidal activity of macrophages and innate immune response mediated by TLR9. This behaviour is one of the mechanisms for circumventing bacterial innate immunity, which relies on the self-degradation of CpG-rich islands by bacterial DNase [57]. None of the isolates selected for this study showed DNase activity, which was confirmed by the absence of a DNase activity zone (Figure 1a). Therefore, the absence of DNase was confirmed in the tested strains, thereby supporting the safety of their use in fermentation. Recent research by Simões et al. [58] reported that *L. paracasei* subsp. *Paracasei* CCMA 1770, *L. pentosus* CCMA 1768, and *L. brevis* CCMA 1762 isolated from fermented olives showed no DNase activity. This study is important for figuring out if probiotics are safe for human consumption.

Haemolysis is a major virulence factor of pathogenic bacteria, facilitating the absorption of iron and potentially leading to anemia in the host [59]. Assessing haemolytic activity (α or β) serves as a common in vitro method for evaluating safety even for bacterial strains classified as Generally Recognized as Safe (GRAS) [14]. Furthermore, our isolates showed no signs of either α- or β-haemolytic activity during cultivation on Columbia sheep blood agar, indicating an absence of haemotoxins (Figure 1b). These findings are consistent with previous studies on *Lactobacillus* strains isolated from fermented foods and goat milk, which similarly reported negative results for haemolytic activity [8]. Additionally, non-haemolytic probiotic strains can exert protective effects against pathogens that induce haemolysis [60]. Moreover, our strains did not show any enzymes linked to virulence, like gelatinase, which adds to the assurance of their suitability for purposes. The lack of gelatinase, DNase, and haemolytic actions in these samples indicates that they could potentially serve as options for incorporation in food fermentation and probiotic practises.

#### 3.4.2. Determination of Antibiotic Resistance

**Figure 2:**
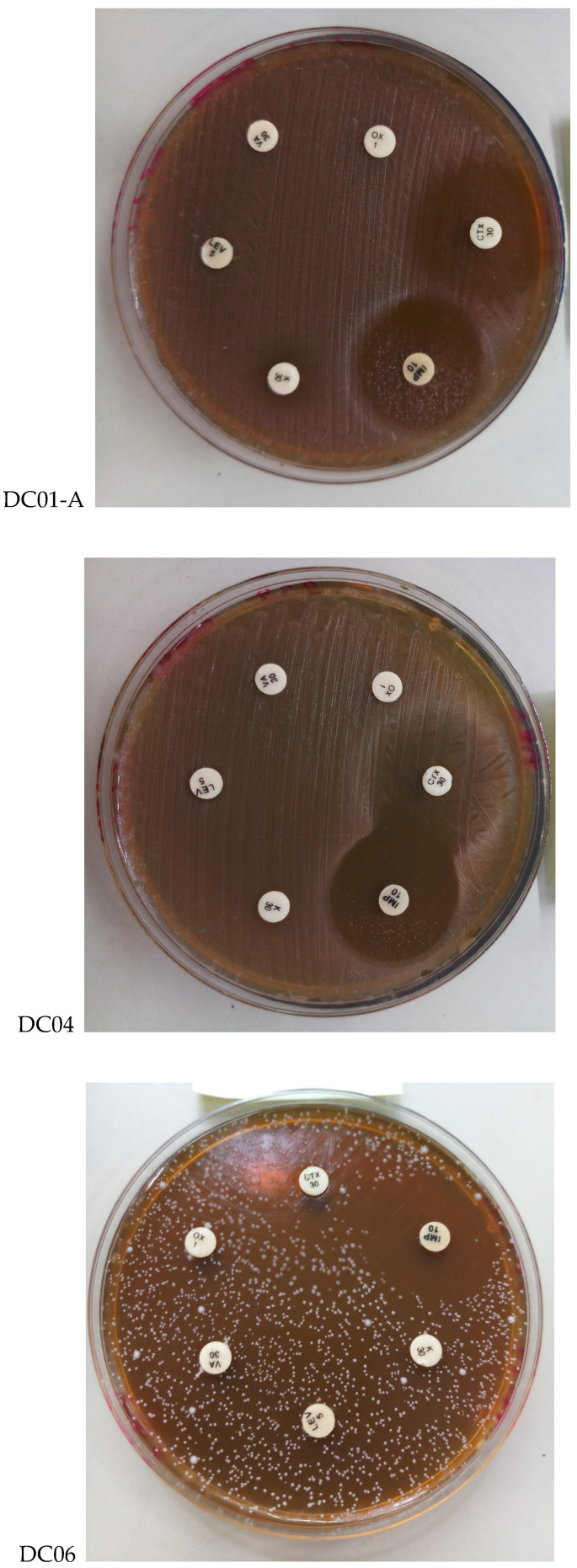
Antibiotic resistance of *Lactobacillus* strains to different antibiotics. DC01-A: *Levilactobacillus brevis*; DC04: *Lactiplantibacillus plantarum*; DC06: *Levilactobacillus brevis*. IMP; Imipenem, K; Kanamycin, LEV; Levromycin, VA; Vancomycin, OX; Oxacillin, CTX; Cefotaxim.

When assessing probiotic bacteria, assessing their antibiotic resistance profiles is essential to ensure their safety for dietary consumption and to avoid the transmission of resistance genes to pathogenic microorganisms [47]. According to probiotic criteria, isolated LAB should not carry antibiotic resistance genes that could contribute to the spread of resistance among human pathogens. In this study, antibiotic susceptibility was determined using the disc diffusion method, and the results, summarized in Table 6 and Figure 2, reveal that all isolates were susceptible to most broad-spectrum antibiotics but resistant to antibiotics primarily targeting Gram-negative bacteria.

These results are consistent with those of previous studies, demonstrating that *Lactobacillus* strains exhibit resistance to vancomycin. This resistance is attributed to the presence of d-Ala-d-lactate in their peptidoglycan structure rather than the typical d-Ala-d-Ala dipeptide [61]. Many *Lactobacillus* species, including *L. casei*, *L. plantarum*, and *L. acidophilus*, exhibit intrinsic resistance not only to glycopeptides but also to other antibiotics such as penicillin G, norfloxacin, ciprofloxacin, and ofloxacin [62]. Moreover, most species of LAB display resistance to aminoglycosides (kanamycin and gentamicin) and quinolones (norfloxacin, ciprofloxacin, and nalidixic acid) [63]. This resistance profile suggests that LAB isolates, despite their resistance to multiple antibiotics, may serve as valuable adjuncts to antibiotic therapy by helping to restore microbial balance more rapidly during treatment [64]. Such applications highlight the importance of selecting probiotic strains that not only exhibit beneficial properties but also maintain sensitivity to antibiotics to minimize the risk of transferring resistance genes to pathogenic bacteria in the human gut. This evaluation is critical to ensure the safety and effectiveness of probiotics in dietary applications, particularly as antibiotic resistance continues to pose a significant public health challenge.

### 3.5. Strain Characteristics Associated with Probiotic Potential

#### 3.5.1. Tolerance of *Lactobacillus* Strains at Different Bile Salt Concentrations

**Figure 3:**
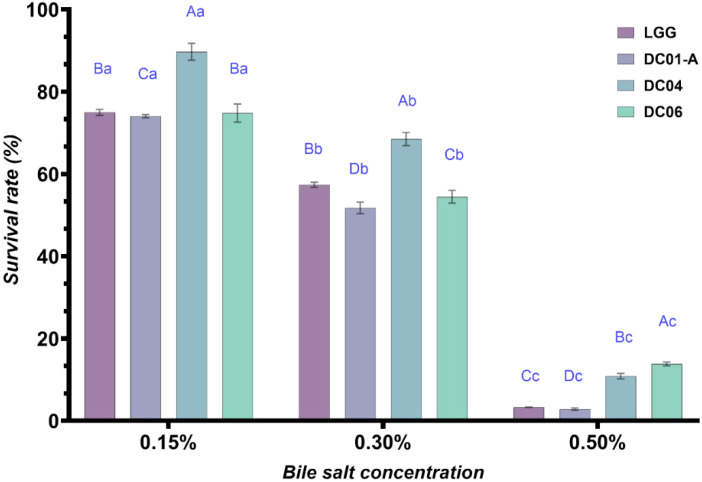
Survival rate (%) of *Lactobacillus* strains at 0.15, 0.30, and 0.50% bile salt. ^a–c^: different lowercase letters in superscript indicate significant differences (*p* < 0.05) among bile salt concentration for same strains. ^A–D^: different uppercase letters in superscript within indicate significant differences (*p* < 0.05) among strains. DC01-A: *Levilactobacillus brevis*; DC04: *Lactiplantibacillus plantarum*; DC06: *Levilactobacillus brevis*.

As probiotics are typically administered orally, their ability to survive the harsh conditions of the gastrointestinal tract is essential. Consequently, their resistance to the low pH of gastric juice and to the presence of bile salts in the small intestine are crucial criteria for selecting probiotic strains [65]. Tolerance to bile salt is a pre-requisite to allow the LAB to colonize and secrete a beneficial effect in the small intestine of the host. The concentration of bile salt used in this work equals the concentration present in human intestine. The relevant physiological concentration of human bile ranges between 0.30 and 0.50% [66]. In this study, all tested strains demonstrated consistent bile tolerance at concentrations between 0.15 and 0.30%. At a concentration of 0.5%, there was a significant reduction (*p* < 0.05) in viable cell numbers, whereas lower concentrations did not affect viability (Table 7, Figure 3).

Previous studies corroborate these findings, showing strain-dependent bile tolerance. Kimoto-Nira et al. [67] reported that *L. brevis* KB290 exhibited enhanced growth in the presence of bile concentrations ranging from 0.15 to 0.50%, which promoted increased cell yield, hydrophobicity, and lactate production. Similarly, *L. plantarum* ATCC 14917 demonstrated tolerance to bile concentrations up to 0.70% [68]. Different mechanisms are used by LAB to protect themselves against the harmful effects of bile salt deconjugation. These include cell envelope remodelling, enhanced bile efflux systems, and the production of bile salt hydrolase (BSH) enzymes [69]. Moreover, transcriptomic and proteomic analyses of *L. salivarius* strains exposed to bile stress revealed adaptations in carbohydrate metabolism, cell surface modifications, and the activation of proteolytic systems [70]. Additionally, probiotics employ antioxidant mechanisms to combat bile-induced oxidative stress [70]. The ability to resist bile salt stress is essential not only for bacterial colonization but also for maintaining a balanced intestinal microbiota [1]. The bile tolerance demonstrated by the LAB isolates in this study suggests that these strains are able to survive in the small intestine and colon, indicating their potential efficacy as probiotic candidates for gastrointestinal applications.

#### 3.5.2. Tolerance of *Lactobacillus* Strains to NaCl, pH, and Phenols

The ability of the isolates to survive in various stressful environments was evaluated. All *Lactobacillus* isolates demonstrated tolerance to salt concentrations of up to 10%, exhibiting significant growth across all tested levels (Table 8). This capacity to grow under elevated salt conditions classifies them as osmotolerant [25]. This osmotolerance is crucial as it enables LAB to maintain metabolic functions and lactic acid production even in high-salt environments, such as those found in the gastrointestinal tract [71]. It also provides a distinct advantage during industrial processing, where probiotic are exposed to osmotic stress caused by fluctuations in solute concentrations, which can impact cellular hydration, survival, and metabolic activities [72]. Additionally, the ability to tolerate NaCl is particularly relevant in the context of incorporating probiotics into fermented foods, many of which contain varying salt levels. Probiotic strains that withstand these conditions are more likely to survive during product formulation, storage, and consumption, ensuring their viability and efficacy [73].

Acid tolerance is a critical attribute for probiotic functionality, as it determines a strain’s ability to survive in the harsh acidic environment of the human stomach. The pH of the human stomach ranges from 1.5 to 4.5, depending on food intake and digestion, with food typically remaining in the stomach for approximately 2–3 h [74]. As shown in Table 8, most strains exhibited growth at pH levels between 3 and 5, although their growth at pH 1.5 was minimal. Such acidic conditions are essential for ensuring that probiotic strains can endure gastric passage and successfully colonize the gastrointestinal tract [75]. LAB are frequently utilized in lactic acid fermentation due to their ability to survive and multiply under acidic environments, such as the stomach, where the pH ranges from 1.5 to 2.5 prior to transit to the gastrointestinal tract [76]. Furthermore, the ability of LAB strains to grow under acidic conditions makes them ideal candidates for use as potential probiotics [77]. Several studies have demonstrated the resistance of LAB to acidic environments. For instance, strains of LAB derived from plant-based fermented food such as kimchi have exhibited significant resistance to simulated gastric juice conditions, with a survival rate of more than 90% [78]. *Lactobacillus* strains isolated from meat products were also found to be tolerant to pH 2.0 [79]. Various mechanisms have been proposed to explain the resistance of LAB to acidic conditions. One such protective mechanism is the secretion of a proton-translocating ATPase, which helps stabilize the internal cellular pH in response to very low external pH [77]. Overall, the ability of LAB to survive and thrive under acidic environments make them highly valuable in lactic acid fermentation and as promising probiotics that can withstand the challenging conditions of the digestive system.

Phenol resistance has been tested as an indicator of isolate survival under intestinal conditions [80]. Phenolic compounds, produced by the deamination of aromatic amino acids by intestinal microbiota, can inhibit bacterial growth and alter the diversity and metabolic activity of gut communities [81]. Phenolic compounds in foods, including oils, beans, fruits, and wines, can vary widely, with concentrations ranging from 0.0002 to 3.6% (*w*/*w*) [82]. Consequently, phenol concentrations of 0.2 and 0.5% were selected to assess the isolates, representing the average phenolic content found in foods. Therefore, phenol tolerance is an interesting issue in the characterization of probiotic strains. In our study, all tested isolates showed substantial growth in MRS medium with 2% phenol, although growth was significantly reduced at 5% phenol (Table 8). These findings align with those of Divisekera et al. [83], who reported that three *Lactobacillus* species were unable to tolerate even 0.5% phenol. LAB strains isolated from fermented foods have shown varying tolerance to phenolic compounds [73]. Additionally, LAB strains with probiotic potential, such as *L. reuteri* and *Pediococcus acidilactici*, have demonstrated tolerance to phenol and bile salts, as well as adherence to ileum epithelial cells [84].

#### 3.5.3. Auto-Aggregation Ability of *Lactobacillus* Strains

**Figure 4:**
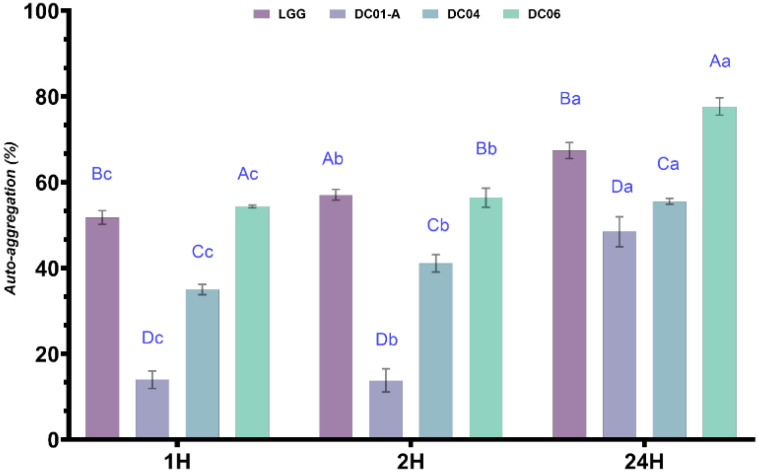
Auto-aggregation percentages of *Lactobacillus* strains. *Lactobacillus* LGG was used as a reference probiotic strain. Data are expressed as mean ± SD (n = 3). ^a–c^: different superscript in lowercase letters represent significant differences (*p* < 0.05) among time for the same strain. ^A–D^: different uppercase letters in superscript indicate significant differences (*p* < 0.05) among strains. DC01-A: *Levilactobacillus brevis*; DC04: *Lactiplantibacillus plantarum*; DC06: *Levilactobacillus brevis*.

In vitro studies were conducted to assess the hydrophobicity and auto-aggregation properties of *Lactobacillus* strains, as these surface characteristics are essential for the adhesion of probiotic bacteria to epithelial cells [85]. The ability to attach to epithelial cells and establish colonies within the gastrointestinal tract is a vital trait of probiotics, promoting their competitive growth and spread within the host environment. This property also enhances the ability of probiotics to outcompete pathogens by forming protective barriers along the intestinal wall [86]. In our study, the isolates exhibited increased auto-aggregation with prolonged incubation times (Figure 4), suggesting their potential for colonizing and adhering to the intestinal epithelium. The results demonstrated significant aggregation across all strains tested. For strain DC01-A, aggregation values increased from 13.91% at 1 h and 13.78% at 4 h to 48.46% at 24 h. Strain DC04 showed higher aggregation, starting at 34.97% at 1 h, rising to 41.09% at 2 h, and reaching 55.52% at 24 h. Similarly, DC06 exhibited strong aggregation, with 54.36% at 1h, 56.40% at 2 h, and 75.57% at 24 h. These values were compared with the reference strain LGG, which showed aggregation levels of 51.78%, 57.07%, and 69.46% at 1 h, 2h, and 24 h, respectively. The auto-aggregation percentages observed for *L. brevis* strains in this research were comparable to those reported for various *Lactobacillus* strains in other studies [87]. According to Wang et al. [88], bacterial strains with aggregation of at least 40% are classified as having good auto-aggregation properties, while those below 10% are considered weak. Conversely, Rahman et al. [89] suggested that a threshold of 70% should be used to define high aggregation capacity. Despite the variability in classification criteria, the strains in our study exhibited substantial aggregation, indicating robust surface adhesion capabilities.

#### 3.5.4. Determination of Cell-Surface Hydrophobicity

The adhesion of bacteria to both abiotic and biotic surfaces is a complex process influenced by various factors, including hydrophobicity, which contributes to non-specific physical interactions that facilitate auto-aggregation and adhesion [42,73]. The BATH assay is a method widely used to evaluate the hydrophobicity and adhesion properties of LAB to surfaces. A higher a polarity of bacterial surfaces correlates with enhanced adhesion to hydrocarbons, reflecting improved hydrophobicity; in particular, adhesion to xylene reflects the hydrophobicity of cell surfaces [90]. Tyfa et al. [91] categorized bacterial strains based on their hydrophobicity: strongly hydrophobic (>50%), moderately hydrophobic (20–50%), and hydrophilic (<20%). This classification has been further investigated in subsequent studies. Probiotic strains with high hydrophobicity (>40%) are preferred for their enhanced ability to adhere to host cells, which is crucial for their beneficial effects [92].

**Figure 5:**
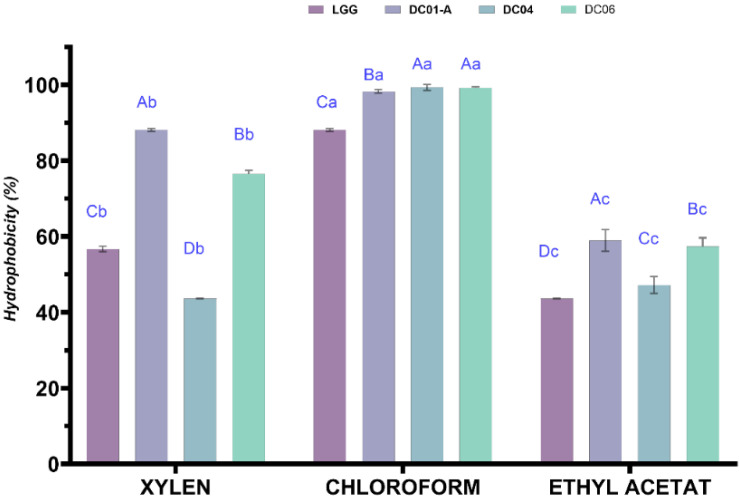
Hydrophobicity percentages of *Lactobacillus* strains. *L. rhamnosus* GG was used as a reference probiotic strain. Data are expressed as mean ± SD (n = 3). ^A–D^: different superscript in uppercase letters represent significant differences (*p* < 0.05) among strains for same solvent. ^a–c^: different small letters in superscript indicate significant differences (*p* < 0.05) among solvent for same strain. DC01-A: *Levilactobacillus brevis*; DC04: *Lactiplantibacillus plantarum*; DC06: *Levilactobacillus brevis*.

According to this criterion, all three strains in this study exhibited strong hydrophobic characteristics (Figure 5); our strains exhibited high adhesion rates to various solvents, demonstrating their potential for strong interaction with cell membranes. Specifically, adhesion to chloroform was observed at 98.26% for DC01-A, 99.30% for DC04, and 99.20% for DC06. With xylene, the adhesion rates were 75.94% for DC01-A, 61.13% for DC04, and 76.52% for DC06. Additionally, the adhesion rates to ethyl acetate were recorded as 58.93% for DC01-A, 47.19% for DC04, and 57.39% for DC06. These results indicate the strains’ significant capability to adhere to hydrophobic surfaces, suggesting their affinity for cell membranes. This shows an amphoteric character and has both Lewis acid and Lewis base properties; compared with commercial strains LGG (88.13%, 56.69%, and 43.65% with chloroform, xylene and ethyl acetate, respectively), these results show that our strains could attach to and colonize intestinal epithelial cells. The lower adhesion of *Lactobacillus* strains to ethyl acetate compared to chloroform and xylene is likely due to differences in solvent polarity and their interactions with bacterial cell surfaces. Ethyl acetate is more polar, which may not promote the same level of interaction with hydrophobic bacterial surfaces as the nonpolar solvents [42]. The specific surface chemistry of the bacteria also plays a role; adhesion is influenced by surface properties such as free energy and topography [73]. Additionally, the molecular structure of ethyl acetate may limit its effectiveness in interacting with hydrophobic surfaces, resulting in lower adhesion rates. Studies suggest that a higher nonpolarity of surfaces leads to greater adhesion and hydrophobicity [90]. Therefore, the lower adhesion percentage for ethyl acetate reflects its polarity and solvent properties, which may not be as consistent with the hydrophobic nature of bacterial cell surfaces compared to chloroform and xylene.

In contrast, the high adherence scores of *Lactobacillus* strains to chloroform may be attributed to the basic properties of the bacterial cells, specifically their characterization as Lewis bases. This basic character is likely related to the presence of carboxylic groups on the microbial surface, which can enhance interactions with monopolar solvents like chloroform. Recent research highlights the significance of bacterial adhesion in various contexts, emphasizing that targeting adhesion mechanisms can serve as a promising anti-virulence strategy against antimicrobial-resistant strains [93]. Understanding these interactions is crucial for evaluating the adhesive properties of probiotics and their effectiveness in applications.

#### 3.5.5. Biofilm Formation

Biofilm formation, a crucial property for probiotic efficacy, was evaluated using an adhesion scale ranging from 0 (no adhesion) to 4 (strong adhesion) [94]. The tested LAB strains demonstrated varying degrees of biofilm-forming ability, with strain DC04 showing strong adhesion (4), DC01-A exhibiting moderate adhesion (3), and DC06 displaying weak adhesion (2). These results were compared with those of the commercial probiotic strain LGG, which was used as a benchmark for biofilm formation (Figure S3). Biofilm formation is considered an important trait for probiotic strains, enhancing colonization and promoting the persistence of LAB on the host’s mucosal surfaces. This capability not only strengthens the probiotic’s ability to adhere to epithelial cells but also prevents the colonization of pathogenic bacteria, thereby providing a protective barrier to the host [94]. Studies have shown that various *Lactobacillus* strains can form biofilms on different surfaces, with strain-specific variations in their biofilm-forming capacity [95]. In addition, the ability of cells to aggregate contributes to biofilm stability and promotes their persistence within the gastrointestinal tract. Proteins, lipoproteins, and polysaccharides found on the bacterial cell surface, as well as in the culture supernatant, are involved in the auto-aggregation process, facilitating interactions between cells [42].

### 3.6. Technological Characterization of Lactobacillus Strains

#### 3.6.1. Alcohol Resistance

The LAB strains demonstrated impressive growth in ethanol concentrations up to 12%, but their tolerance did not exceed this concentration (Table 9), while certain LAB strains LAB such as *L. buchneri* NRRL and *L. casei* typically exhibited high ethanol resistance, with some reaching 18% [96]. In contrast, similar studies have observed limitations in certain strains. Araque et al. [97] found that *L. brevis* maintained viability up to 5% ethanol, with a decrease in population at 10%, and similar ethanol sensitivity was observed for *L. plantarum* [47]. In particular, Liu [98] further reported that *L. buchneri* NRRL exhibited growth inhibition and decreased biosynthesis under higher ethanol stress. Selecting ethanol-tolerant strains is critical for applications requiring resilience to harsh environments and alcohol production [44].

#### 3.6.2. Hydrogen Peroxide (H_2_O_2_) Resistance

For the LAB strains, as expected, increasing the concentration of H_2_O_2_ resulted in reduced bacterial growth (Table 9). This inhibition is primarily due to oxidative stress, which causes damage DNA through oxidizing bases and compromising cell membranes via lipid peroxidation [99]. Additionally, oxidative stress may also impair proteins through cysteine oxidation, leading to dysfunctional crosslinked molecules [100]. LAB, including *Lactobacillus* strains, are particularly susceptible to H_2_O_2_ due to the absence of these proteins, which are essential for decomposing hydrogen peroxide into water and oxygen [101]. However, certain LAB can adapt by expressing heme- and manganese-dependent catalases or shifting to respiratory metabolism, enhancing their oxidative stress tolerance [100]. In particular, *L. casei* IGM394 plays a crucial role in H_2_O_2_ breakdown through the activity of NADH peroxidase [102]. The ability to withstand H_2_O_2_ is a fundamental trait of probiotic organisms used in industries, as it not only supports bactericidal activity but also extends product shelf life through enhanced microbial stability.

#### 3.6.3. Growth Response to Temperature Tolerance

The isolates demonstrated growth within a temperature range of 10 °C to 37 °C (Table 9). Although the strains could not survive at 50 °C, both strains DC4 and DC6 demonstrated the ability to proliferate at 45 °C, aligning with the typical growth conditions for *Lactobacillus* species [25]. Their ability to proliferate at 37 °C supports their potential as probiotics, maintaining active metabolism within the human gastrointestinal tract [103]. Additionally, tolerance to higher temperatures enhances growth rates and lactic acid production during fermentation while minimizing contamination risks [53]. Moreover, Mbye et al. [104] reported that certain LAB can endure high temperatures ranging from 45 to 80 °C, showcasing their resilience to extreme thermal conditions. These findings are consistent with the existing literature, as the ability to grow at moderate temperatures is typical of many LAB strains. Furthermore, since goat milk butter fermentation takes place at room temperature (around 25 °C), it is expected that all the tested strains would thrive under these conditions, ensuring optimal metabolic activity and successful fermentation.

#### 3.6.4. Autolytic Activity

**Figure 6:**
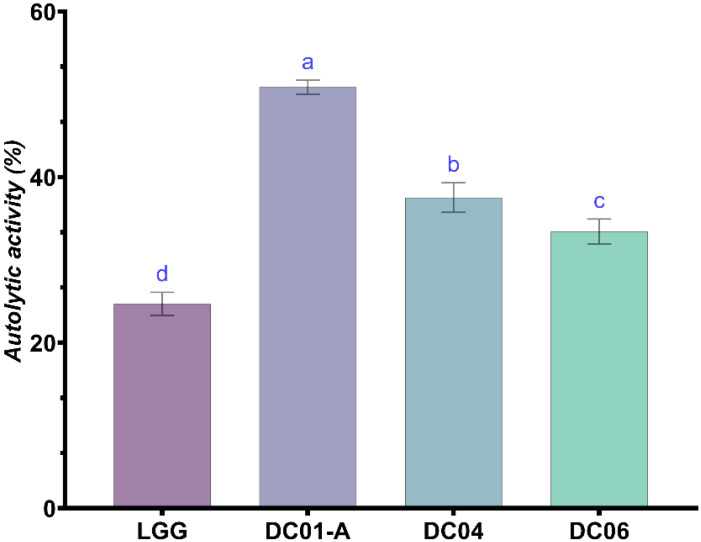
Autolytic activity (%) of *Lactobacillus* strains. Data are expressed as mean ± SD (n = 3). ^a–d^: different small letters in superscript indicate significant differences (*p* < 0.05) among strains. DC01-A: *Levilactobacillus brevis*; DC04: *Lactiplantibacillus plantarum*; DC06: *Levilactobacillus brevis*.

Autolytic activity is a notable characteristic of LAB, characterized by the release of intracellular enzymes, such as lipase and protease, that enhance the sensory and textural qualities of fermented products. This process is driven by the catalytic action of peptidoglycan hydrolase on the bacterial cell wall structure, resulting in the breakdown of cellular components and enzyme release [47]. The release of these enzymes plays a crucial role in cheese ripening, enhancing flavour development and texture [105]. As shown in Figure 6, the isolates in this study demonstrated medium to high autolytic activity at 50.86, 37.53, and 33.42% for DC01-A, DC04, and DC06, respectively, compared to the reference strain LGG, which exhibited 24.69% autolysis. The degree of autolysis in LAB is strain-dependent, as demonstrated in several studies. Barzegar et al. [47] investigated the autolytic properties of *Lactobacillus* strains isolated from local Iranian cheese. Their study found that the autolytic activity of eight isolates ranged from 10 to 38%. Similarly, Wei-Zhen and Yi [106] found that the autolysis of *Lactococcus lactis* MG1363 was influenced by different growth inhibitors, indicating a strain-specific response. Al-Saleh et al. [107] observed variations in autolysis ability and the presence of autolytic enzymes among LAB strains. This was further supported by Pang et al. [108], who identified the role of N-acetylmuramidase in the autolysis of *L. delbrueckii* subsp. *bulgaricus* and *L. sakei*, suggesting a strain-specific mechanism.

### 3.7. Biological Activities of Lactobacillus Strains

#### 3.7.1. Antibacterial Activity

LAB are receiving increasing attention for their potential as bioprotective cultures in food preservation, particularly in response to the “Clean Label” trend and growing concerns about antibiotic resistance [109]. LAB produce various antimicrobial substances, including organic acids, hydrogen peroxide, and bacteriocins, which can inhibit both spoilage organisms and foodborne pathogens [110]. Bacteriocins, proteinaceous toxins produced by LAB, have shown effectiveness against numerous pathogens and spoilage bacteria, contributing to the extended shelf life and safety of raw meats, dairy, and fermented foods. These bacteriocins vary in molecular weight from 7.0 to 15.0 kDa, and their activity is influenced by environmental factors such as heat, pH, and enzymes [111].

**Figure 7:**
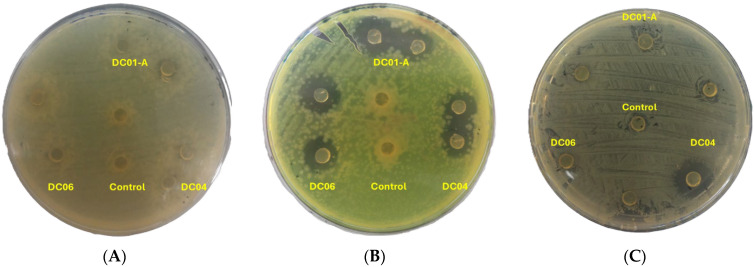
Antibacterial activity of lactic acid bacteria. (**A**) *Escherichia coli* ATCC10536, (**B**) *Pseudomonas aeruginosa* ATCC27853, and (**C**) *Staphylococcus aureus* ATCC25923. DC01-A: *Levilactobacillus brevis*; DC04: *Lactiplantibacillus plantarum*; DC06: *Levilactobacillus brevis*.

The antimicrobial activity of LAB isolates in this study further underscores their potential for food safety applications. As shown in Table 10 and Figure 7, isolate DC4 exhibited significant antimicrobial effects against *S. aureus*, with an inhibition zone of 22.00 mm. This isolate also exhibited moderate inhibition against *P. aeruginosa* (15.60 mm) and *E. coli* (12.30 mm). Other isolates varied in their effectiveness, with DC1-A and DC6 all showing inhibition against *P. aeruginosa* (19.72 and 20.34 mm, respectively) and *E. coli* (11.32 and 7.25 mm, respectively). These results suggest that the LAB strains produced antimicrobial compounds capable of inhibiting pathogen growth. Prior research has shown that *L. plantarum* can effectively inhibit the growth of pathogens such as *Listeria monocytogenes*, *S. aureus*, *E. coli* O157:H7, *P.aerogenosa*, and several species of *Salmonella* [112]. Additionally, some *L. brevis* strains have demonstrated the ability to inhibit biofilm formation by pathogens, which is significant for controlling persistent infections and contamination [113]. Co-cultivation experiments have revealed that *L. plantarum* can sustain high viable cell counts while simultaneously reducing the population of harmful pathogens [11]. These findings suggest that the LAB strains have potential as bio-control agents against foodborne pathogens in various food applications.

#### 3.7.2. Antioxidant Activity

**Figure 8:**
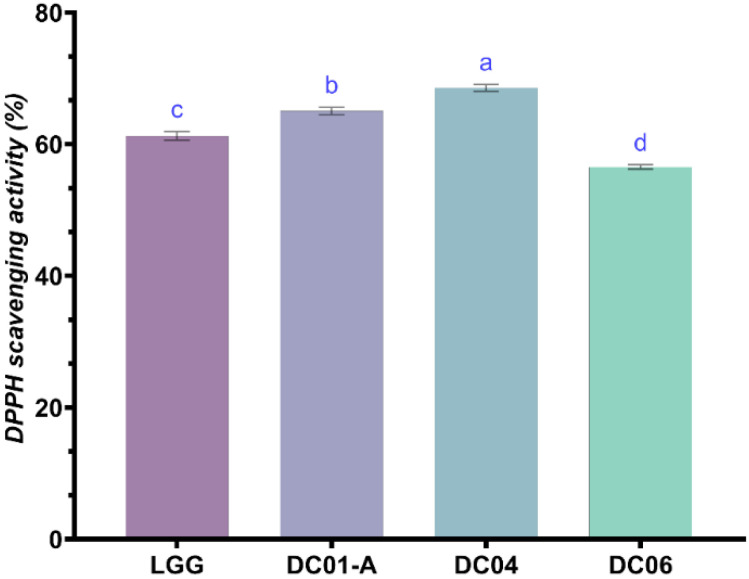
Antioxidant activity of *Lactobacillus* strain cell-free supernatant evaluated using DPPH (%). ^a–d^: different small letters in superscript indicate significant differences (*p* < 0.05) among strains. DC01-A: *Levilactobacillus brevis*; DC04: *Lactiplantibacillus plantarum*; DC06: *Levilactobacillus brevis*.

In our study, we investigated the antioxidant activity of three *Lactobacillus* isolates, with a particular focus on their DPPH radical scavenging abilities. The results show that strain DC04 exhibited the highest DPPH scavenging activity with 68.56%, compared to LGG, which demonstrated 61.28% scavenging activity; in addition, DC01A and DC06 showed relatively high activity up to 65.1% and 56.6%, respectively (Figure 8). These results suggest that certain LAB strains have significant antioxidant abilities. The observed antioxidant potential of our isolates can be attributed to the production of various bioactive compounds during fermentation. A study by Kim et al. [114] reported that the *Levilactobacillus brevis* KU15147 strain exhibited significant DPPH scavenging activity, supporting its role in promoting health through antioxidant effects. Numerous studies have investigated the DPPH scavenging ability and reducing power of CFS of LAB [115]. For instance, LAB strains exhibited similar DPPH scavenging activities of up to 32.9% [116], while Qadi et al. [11] demonstrated that LAB isolated from plant-based sources exhibited significant antioxidant properties, including the inhibition of DPPH and FRAP radical formation.

The antioxidant properties of CFS were attributed to various metabolites, including organic acids, fatty acids, and proteinaceous compounds [117]. Specifically, anserine, GABA, acetic acid, lactic acid, and other metabolites were identified as the bioactive compounds contributing the most to the highest antioxidant activity in the LAB supernatant [11]. Previous research has also linked LAB antioxidant activity to the production of cell surface compounds such as exopolysaccharides and lipoteichoic acid, which can effectively scavenge free radicals [118]. This finding highlights the importance of researching indigenous LAB strains for their potential health benefits. Our results are consistent with the existing literature emphasizing the role of LAB in the production of bioactive metabolites that increase antioxidant activity.

## 4. Conclusions

The isolation and identification of LAB from traditional Dhan butter revealed a predominance of *Levilactobacillus brevis* and *Lactiplantibacillus plantarum*. These LAB species are recognized for their probiotic properties and their essential role in fermentation, suggesting that they may contribute to the distinctive flavour and potential health benefits of traditional Dhan butter. Safety assessments confirmed that none of the isolated LAB strains posed any health risks, as they exhibited no gelatinase, haemolytic, or DNase activity. The isolates also displayed impressive resistance to bile salts, NACL, varied pH levels, and phenolic compounds, along with significant hydrophobicity and strong auto-aggregation capabilities. The technological characterization of the LAB strains reveals that the strains exhibited strong growth in ethanol concentrations of up to 12%, and thrived within a temperature range of 10 to 37 °C. While higher concentrations of H_2_O_2_ adversely affected growth, the medium to high autolytic activity (33.42–50.86%) indicates substantial autolytic capabilities that can enhance the sensory qualities of fermented products. In the term of biological activities, the strains showed antibacterial activity against *E. coli*, *P. aeruginosa*, and *S. aureus*, along with antioxidant properties measured by DPPH assays. In summary, our results underscore the unique microbial profile of traditional Dhan butter and its potential health-promoting properties, paving the way for future research into its functional applications in nutrition and health.

## Figures and Tables

**Table 1:** Antibiotics used in this study.

Antibiotic	Concentration	Symbol
Nalidixic acid	30 µg	NA
Ampicillin	10 µg	AMP
Cefotaxim	30 µg	CTX
Colestin	10 µg	CL
Gentamycin	10 µg	GEN
Imipenem	10 µg	IMP
Levromycin	5 µg	LEV
Kanamycin	30 µg	K
Oxacillin	1 µg	OX
Penicillin	10 µg	P
Rifampicin	5 µg	RIF
Tobramycin	10 µg	TOB
Vancomycin	30 µg	VA

**Table 2:** Preliminary tests on physiological and biochemical characteristics of *Lactobacillus* strains isolated from Dhan.

Strains	Gram	Forme	Catalase	Mobility	VP	Cit	Gaz from Glu
DC01-A	+	Bacille	-	-	-	-	+
DC04	+	Bacille	-	-	-	-	+
DC06	+	Bacille	-	-	-	-	+

+, positive; -, negative; (VP), Vosges–Proskauer; Cit, citrate fermentation; Glu, glucose fermentation.

**Table 3:** Identification of *Lactobacillus* strains by API 50 CHL.

Isolates	Species Identified by API Test
DC01-A	*Levilactobacillus brevis*
DC04	*Lactiplantibacillus paraplantarum*
DC06	*Levilactobacillus brevis*

**Table 4:** Molecular identification of *Lactobacillus* strains isolated from Dhan using NCBI (nt_prok database).

Isolates	Top-Hit Taxon	GenBank Accession No	Identity (%)	Query Cover (%)
DC01-A	*Levilactobacillus brevis strain* 6525	MT515953	99.93	99.00
DC04	*Lactiplantibacillus plantarum strain* ZYL14	PP215884	99.86	100
DC06	*Levilactobacillus brevis strain* 3379	MT512165	99.00	99.86

**Table 5:** Percent sequence similarity between *Lactobacillus* strains and type strains of validly published prokaryotic names (available online at http://eztaxon-e.ezbiocloud.net/ accessed on 10 June 2024).

Isolates	Top-Hit Taxon	Top-Hit Strain	Similarity (%)	Completeness (%)
DC01-A	*Levilactobacillus brevis*	ATCC 14869	99.45	97.5
DC04	*Lactiplantibacillus plantarum*	ATCC 14917	99.79	100.0
DC06	*Levilactobacillus brevis*	ATCC 14869	99.38	98.1

**Table 6:** Antibiotic sensitivity of *Lactobacillus* strains, as measured by diameter of inhibition zones.

Antibiotic	DC01-A	DC04	DC06
Nalidexic acid	R	R	R
Ampicillin	R	S (26.1)	R
Cefotaxim	S (25.8)	S (22.0)	S (26.0)
Colistin	R	R	R
Gentamycin	S (18.4)	R	S (18.2)
Imipenem	S (26.6)	S (23.2)	S (26.5)
Levromycin	R	R	R
Kanamycin	R	R	R
Oxacillin	R	R	R
Penicillin	R	R	R
Rifampicin	S (18.3)	S (22)	S (20.4)
Tobramycin	S (10.7)	R	S (11.3)
Vancomycin	R	R	R

Zone of inhibition (mm); R, resistant; S, susceptible. DC01-A: *Levilactobacillus brevis*; DC04: *Lactiplantibacillus plantarum*; DC06: *Levilactobacillus brevis*.

**Table 7:** Effect of bile salt concentration on growth of *Lactobacillus* strains at 37 °C.

	Bile Concentration (%)			
Isolates	0	0.15	0.3	0.5
DC01-A	8.66 × 10^6^ ± 0.03 ^Aa^	6.40 × 10^6^ ± 0.02 ^Ab^	4.40 × 10^6^ ± 0.09 ^Bc^	2.50 × 10^5^ ± 0.02 ^Bd^
DC04	2.00 × 10^6^ ± 0.02 ^Ca^	1.94 × 10^6^ ± 0.02 ^Cb^	1.50 × 10^6^ ± 0.01 ^Dc^	2.03 × 10^5^ ± 0.03 ^Ca^
DC06	7.10 × 10^6^ ± 0.04 ^Ba^	5.30 × 10^6^ ± 0.03 ^Bb^	3.80 × 10^6^ ± 0.03 ^Cc^	2.80 × 10^5^ ± 0.03 ^Ad^
LGG	8.90 × 10^7^ ± 0.10 ^Aa^	6.60 × 10^7^ ± 0.08 ^Ab^	5.06 × 10^7^ ± 0.05 ^Ac^	2.97 × 10^7^ ± 0.07 ^Ad^

The mean values, in CFU/mL, along with their standard deviations (n = 3) were used to express the data. ^a–d^: different lowercase letters in superscript within the same row indicate significant differences (*p* < 0.05) among bile salt concentration. ^A–D^: different uppercase letters in superscript within the column indicate significant differences (*p* < 0.05) among strains. DC01-A: *Levilactobacillus brevis*; DC04: *Lactiplantibacillus plantarum*; DC06: *Levilactobacillus brevis.*

**Table 8:** Tolerance characteristics of *Lactobacillus* strains.

Strain	NaCl %					pH			Phenol %	
	2	3	4	6.5	10	1.5	3	5	2	5
DC01-A	+++	+++	++	+	+	-	+	++	++	+
DC04	+++	+++	++	+	+	-	+	++	++	+
DC06	+++	+++	++	+	+	-	+	++	++	+

-: No growth; +: weak growth; ++: moderate growth; +++: strong growth. DC01-A: *Levilactobacillus brevis*; DC04: *Lactiplantibacillus plantarum*; DC06: *Levilactobacillus brevis.*

**Table 9:** Technological properties of *Lactobacillus* strain.

	Isolates		
Characteristics	DC01-A	DC04	DC06
Alcohol			
3%	+++	+++	+++
6%	++	++	++
12%	+	+	+
15%	-	-	-
Growth			
6 °C	+	+	+
10 °C	++	++	++
25 °C	+++	+++	+++
37 °C	+++	+++	+++
45 °C	-	+	+
50 °C	-	-	-
H_2_O_2_			
0.4 mM	0.308 ± 0.022	0.307 ± 0.018	0.415 ± 0.029
0.7 mM	0.102 ± 0.041	0.158 ± 0.055	0.122 ± 0.065
1.0 mM	0.040 ± 0.018	0.098 ± 0.046	0.066 ± 0.025

Maximum growth is represented by three positive signs (+++), high growth by two positive signs (++), weak growth by a single positive sign (+), and no growth by a negative sign (-). DC01-A: *Levilactobacillus brevis*; DC04: *Lactiplantibacillus plantarum*; DC06: *Levilactobacillus brevis.*

**Table 10:** Antibacterial activity of *Lactobacillus* strains against pathogenic bacteria using the disc diffusion method.

Strains	*E. coli*	*P. aeruginosa*	*S. aureus*
	Zone of Inhibition (mm)		
DC01-A	11.32 ± 0.62 ^b^	19.72 ± 2.50 ^b^	7.70 ± 0.60 ^c^
DC04	12.30 ± 0.42 ^a^	15.60 ± 0.60 ^c^	22.00 ± 1.00 ^a^
DC06	7.25 ± 0.60 ^c^	20.34 ± 1.52 ^a^	20.34 ± 1.52 ^b^

The mean values are presented along with their respective standard deviations (n = 3). ^a–c^ Significant differences among the values within the same column are indicated by different superscript letters (*p* < 0.05). DC01-A: *Levilactobacillus brevis*; DC04: *Lactiplantibacillus plantarum*; DC06: *Levilactobacillus brevis.*

## Data Availability

The original contributions presented in the study are included in the article/Supplementary Materials, further inquiries can be directed to the corresponding author.

## Supplementary Materials

The following supporting information can be downloaded at https://www.mdpi.com/article/10.3390/foods13233781/s1: Figure S1: Sampling site location. Illustration of map depicting the geographical location of the Sfisifa region of Naama in western Algeria (32°44′ N 0°52′ W). Figure S2: Phylogenetic tree based on the 16S rRNA gene sequences of *Lactobacillus* strains (marked with a blue triangle). *Escherichia coli* JCM1649 was used as an outgroup reference strain. The scale bar represents 0.02 nucleotide substitution per site. Figure S3: Biofilm formation of *Lactobacillus* strains. Biofilm adhesion levels were quantified on a scale from 0 (no adhesion) to 4 (strong adhesion). DC01-A: *Levilactobacillus brevis*; DC04: *Lactiplantibacillus plantarum*; DC06: *Levilactobacillus brevis.*
