# Supplementary material for: Evaluation of Probiotic Potential and Functional Properties of Lactobacillus Strains Isolated from Dhan, Traditional Algerian Goat Milk Butter
Source: Foods. 2024 Nov 25;13(23):3781. doi: 10.3390/foods13233781 (PMC11640680; doi:10.3390/foods13233781)
Supplement: Supplementary file 1 [file foods-13-03781-s001.zip › foods-3331815-supplementary.pdf]

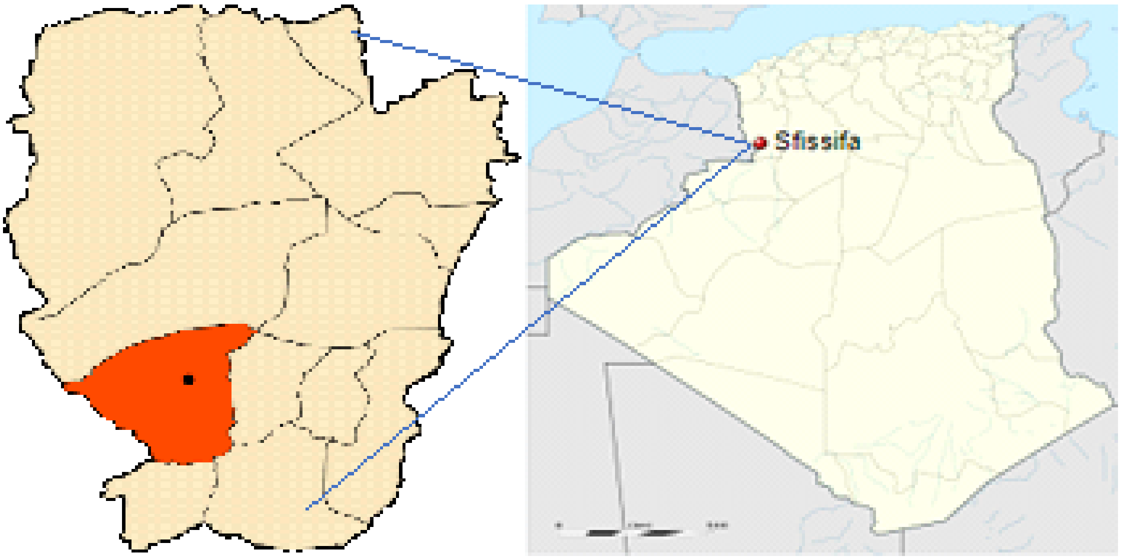

Figure S1. Sampling site location. Illustration of the map depicting the geographical location of the Sfisifa region of Naama in western Algeria (32° 44′ N 0° 52′ W).

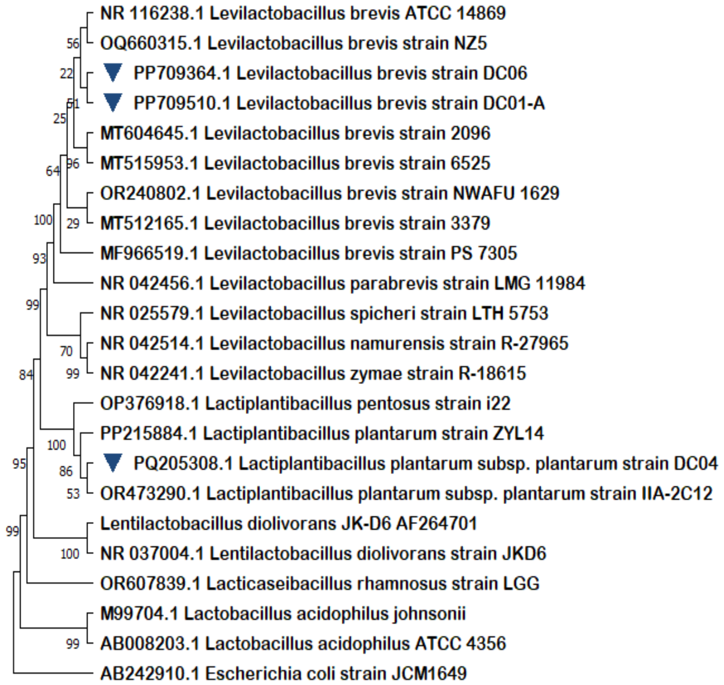

Figure S2. Phylogenetic tree based on the 16S rRNA gene sequences of *Lactobacillus* strains (marked with a blue triangle). *Escherichia coli* JCM1649 was used as an outgroup reference strain. The scale bar represents 0.02 nucleotide substitution per site.

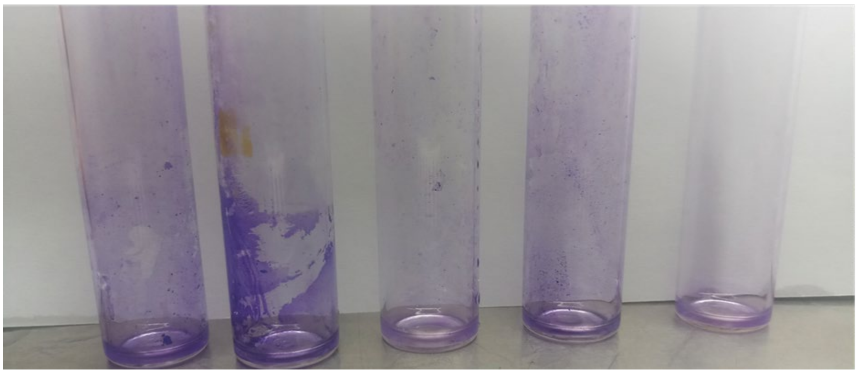

DC01-A (3) DC04 (4) DC06 (2) LGG (3) Control (0)

Figure S3. Biofilm Formation of *Lactobacillus* strains. Biofilm adhesion levels were quantified on a scale from 0 (no adhesion) to 4 (strong adhesion). DC01-A: *Levilactobacillus brevis*; DC04: *Lactiplantibacillus plantarum*; DC06: *Levilactobacillus brevis*.
